# Bioinformatics Analysis of the Prognostic and Biological Significance of ZDHHC-Protein Acyltransferases in Kidney Renal Clear Cell Carcinoma

**DOI:** 10.3389/fonc.2020.565414

**Published:** 2020-12-08

**Authors:** Zhuang Liu, Chang Liu, Mingming Xiao, Yamei Han, Siyue Zhang, Bo Xu

**Affiliations:** ^1^ Department of Biochemistry and Molecular Biology, Key Laboratory of Breast Cancer Prevention and Therapy, Ministry of Education, Tianjin Medical University Cancer Institute and Hospital, National Clinical Research Center for Cancer, Key Laboratory of Cancer Prevention and Therapy, Tianjin, Tianjin’s Clinical Research Center for Cancer, Tianjin, China; ^2^ Department of Microbiology, School of Laboratory Medicine, Tianjin Medical University, Tianjin, China; ^3^ Center for Intelligent Oncology, Chongqing University Cancer Hospital, Chongqing University School of Medicine, Chongqing, China

**Keywords:** kidney renal clear cell carcinoma, ZDHHC-protein acyltransferases, prognosis, immune cell infiltration, pathway analysis

## Abstract

ZDHHC-protein acyltransferases (ZDHHCs) are a family of 23 signature Asp-His-His-Cys (DHHC) domain-containing enzymes that mediate palmitoylation by covalent attachment of the 16-carbon fatty acid palmitate to thiol groups of specific cysteine residues in substrate proteins. Emerging evidence has shown abnormal expression of ZDHHCs in a variety of disease states, including cancer. Kidney renal clear cell carcinoma (KIRC) is the eighth most common type of cancer, which accounts for the majority of malignant kidney tumors. However, there are currently no effective therapeutic targets or biomarkers for clinical treatment and prognosis in KIRC. In this study, we first analyzed the expression pattern of the 23 ZDHHCs in KIRC using TCGA and GEPIA database, and found that the expression of ZDHHC2, 3, 6, 14, 15, 21, and 23 was significantly down-regulated whereas the expression of ZDHHC9, 17, 18, 19 and 20 was significantly up-regulated in KIRC patient tissues vs. normal tissues. And the expression of ZDHHC2, 3, 6, 9, 14, 15, and 21 in tumors decreased with the increase of the pathological stage of KIRC patients. Notably, KIRC patients with decreased expression of ZDHHC3, 6, 9, 14, 15, 17, 20, 21, 23 and increased expression of ZDHHC19 were significantly associated with poor prognosis. Further, we found that there was a significant correlation between ZDHHC3, 6, 9, 14, 15, 17, 19, 20, 21, 23 expressions and immune cell infiltration. Besides, high mRNA expression was the most common type of gene alteration and there was a high correlation among the expression of ZDHHC6, 17, 20 and 21. Finally, function prediction indicated that the immune or metabolic disorders or the activation of oncogenic signaling pathways caused by abnormal expression of these ZDHHCs may be important mechanisms of tumor progression and poor prognosis in patients with KIRC. Our results may provide novel insight for identifying tumor markers or molecular targets for the treatment of KIRC.

## Introduction

Kidney renal clear cell carcinoma (KIRC) is one of the eight most common cancer types, accounting for 70%–80% of renal cell carcinoma (1). Approximately 210,000 new cases are diagnosed worldwide each year (2). Although breakthroughs have been made in the molecular mechanisms and treatment strategies for KIRC in recent years, the prognosis of KIRC patients is still poor, especially for patients with the late clinical stage (3). Studies show that patients with stage I KIRC have a five-year disease-specific survival of about 80%–95%, whereas the five-year disease-specific survival rate of patients with IV KIRC drops sharply to less than 10% (4). Therefore, there is an urgent need to identify promising novel prognostic biomarkers and therapeutic targets, which will help clinicians choose appropriate therapeutic targets and drugs and more accurately predict the long-term prognosis of KIRC patients.

ZDHHC-protein acyltransferases (ZDHHCs) are a family of signature Asp-His-His-Cys (DHHC) domain-containing enzymes that mediate palmitoylation by covalent attachment of the 16-carbon fatty acid palmitate to thiol groups of specific cysteine residues in substrate proteins (5). In humans, the ZDHHC family has been identified to contain 23 members (ZDHHC1-24 skipping ZDHHC10) that play important roles in protein localization, accumulation, secretion, stability, and function (6). Emerging evidence has shown that abnormal expression of ZDHHCs is involved in tumorigenesis and metastasis of various cancers, which seriously affects the treatment and prognosis of cancer patients (7). For example, the low expression of ZDHHC2 in hepatocellular carcinoma was closely related to poor over survival and disease-free survival of patients (8). Elevated expression of ZDHHC3 correlated with enhanced carcinoma growth and diminished patient survival in breast cancer (9). ZDHHC9 inactivation favored NRAS-driven leukemia treatment (10) and enhanced immunotherapy effects for breast cancer (11). Decreasing ZDHHC20 expression increases tumor cell sensitivity to EGFR tyrosine kinase inhibitors (12).

However, the expression pattern, prognostic value, and biological function of ZDHHCs have not been elucidated in KIRC. In this study, we conducted a comprehensive bioinformatics analysis of the expression of ZDHHCs in KIRC. Then, the potential of differentially expressed ZDHHCs to be used as therapeutic targets and prognostic biomarkers was evaluated. Further, function prediction was performed to investigate the potential functions and associated pathways of 10 differentially expressed ZDHHCs. Our study may provide more data to help clinicians choose appropriate therapeutic targets and drugs and more accurately predict the long-term prognosis of KIRC patients.

## Materials and Methods

### Databases and Web Platforms (TCGA, GEPIA, Timer, cBioPortal, STRING, GeneMANIA, GSCALite)

In our study, RNA-Seq data of ZDHHCs in 538 kidney renal clear cell carcinoma (KIRC) tissue samples and 72 normal kidney tissue samples were extracted from The Cancer Genome Atlas (TCGA) database (https://portal.gdc.cancer.gov/) for gene expression analysis and gene set enrichment analysis (GSEA).

GEPIA (Gene Expression Profiling Interactive Analysis, http://gepia.cancer-pku.cn/) was developed at Peking University and is used to analyze differential gene expression, correlation, and patient prognosis based on TCGA and the Genotype-Tissue Expression (GTEx) projects, using a standard processing pipeline (13). In this study, we performed gene expression analysis, pathological stage analysis, prognostic analysis and correlation analysis of ZDHHCs using the **“**KIRC**”** dataset in GEPIA. The parameter **“**Match TCGA normal and GTEx data**”** was set and Student**’**s t test was used to generate a p value for gene expression analysis. The method for gene expression analysis among different pathological stage is one-way ANOVA, using pathological stage as variable for calculating differential expression. Prognostic analysis was performed using a Kaplan–Meier curve and the group cutoff choice was the median. Pearson correlation coefficient was chosen for gene correlation analysis. The p value cutoff was 0.05.

Timer (https://cistrome.shinyapps.io/timer/) provides a user-friendly web interface for dynamic analysis and visualization associations between immune infiltrates and a wide spectrum of factors including gene expression and clinical outcomes across 23 cancer types from TCGA (14). In this study, we evaluated the correlation between differentially expressed ZDHHCs and the infiltration of immune cells using **“**Gene module**”** and the **“**KIRC**”** dataset. Spearman correlation coefficient was chosen for this correlation analysis and a value of P < 0.05 was considered statistically significant.

STRING (Search Tool for the Retrieval of Interacting Genes/proteins, https://string-db.org/) is designed to collect, score, and integrate all public sources of protein-protein interaction (PPI) information, and further calculations are used to construct PPI networks and predict potential interactions (15). In our research, we constructed a PPI network to explore the interaction among differentially expressed ZDHHCs.

The cBioPortal (http://cbioportal.org) provides a web resource for exploring, visualizing, and analyzing multidimensional cancer genomics data based on TCGA database (16). In this study, the data of ZDHHC genetic alterations were obtained from cBioPortal. 538 KIRC samples were selected (TCGA, firehose legacy) for analysis. mRNA expression z scores (RNA Seq V2 RSEM) were obtained using a z score threshold of **±**2.0 and protein expression z scores (RPPA) were obtained using a z score threshold of **±**2.0.

GeneMANIA (http://genemania.org) is a user-friendly web site that can be used to accurately predict the function of the genes submitted and find functionally similar genes using a wealth of genomics and proteomics data (17). In this study, co-expression and interaction analyses of differentially expressed ZDHHCs was performed using GeneMANIA.

GSCALite (http://bioinfo.life.hust.edu.cn/web/GSCALite/) is an available and web-based analysis platform for the gene sets in 32 cancer types from TCGA (18). In this study, GSCALite was used to provide miRNA network analysis of KIRC samples using **“**TCGA KIRC**”** dataset.

### GSEA Method

GSEA (gene set enrichment analysis) reveals many common biological pathways, and this method derives its power by focusing on groups of genes that share common biological function, chromosomal location, or regulation (19, 20). In this study, GSEA v4.0.3 software was used to identify the potential underlying mechanisms of differentially expressed ZDHHCs on the pathogenesis and prognosis of KIRC (TCGA). The V7.0.Gene set in the gene sets database and 1000 for the number of permutations were selected for each analysis.

### Statistical Analysis

**Figure 1:**
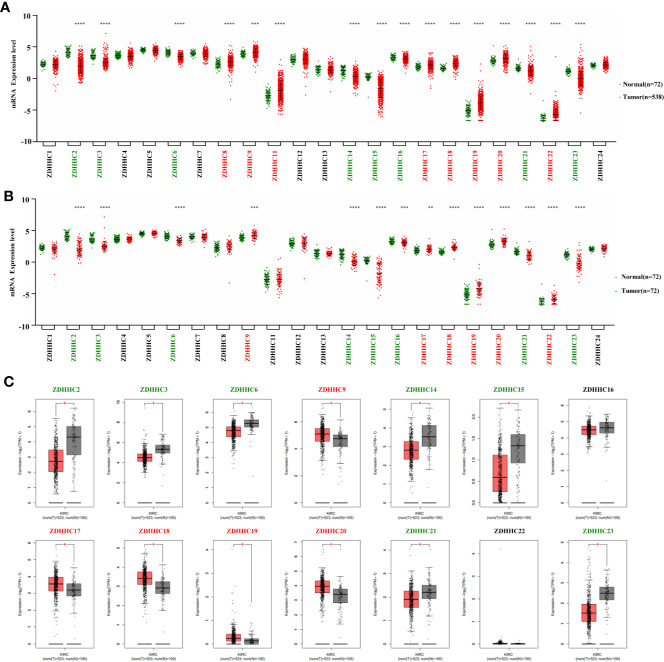
Expression pattern of ZDHHCs in kidney renal clear cell carcinoma (KIRC). **(A)** Expression pattern of ZDHHCs in 538 KIRC patient tissues and 72 normal tissues (TCGA) and statistical differences between two groups were determined by Student’s test for equal variance or Welch t-test for unequal variance (normal distribution) and the Mann-Whitney U-test (non-normal distribution). **(B)** Expression pattern of ZDHHCs in 72 paired KIRC tissues and corresponding adjacent normal tissues (TCGA) and statistical differences between two groups were determined using the paired t-test. **(C)** Expression pattern of ZDHHCs in KIRC patients and normal tissues from GEPIA. statistical differences between the two groups were determined by Student’s t-test and the p value cutoff was 0.05. The green font represents down-regulation, the red font represents up-regulation, and the black font represents no expression difference. * < 0.05; ** < 0.01; *** < 0.001; **** < 0.0001.

SPSS 20.0 (IBM, SPSS Inc., Chicago, IL) software was used to perform the statistical analyses in this study. In **Figure 1A**, if two groups were with normal distribution, then we used the standard Student**’**s test for equal variance or Welch t-test for unequal variance. Otherwise, we used the Mann-Whitney U-test (non-normal distribution). In **Figure 1B**, the paired t-test was used to determine statistical differences between the paired two groups. One-way ANOVA was used to determine gene expression difference among the pathological stage of KIRC patients in GEPIA. Survival curves were generated from Kaplan-Meier Plotter and GEPIA with HR and P-value or Cox P-value using log-rank test. The correlation between gene expression and immune cell infiltration (Timer) was assessed based on statistical significance and Spearman**’**s correlation. A value of P < 0.05 was considered statistically significant. For the GSEA method, the nominal p-value (NOM p < 0.05) and false discovery rate (FDR q < 0.25) were used to determine significantly enriched gene sets.

## Results

### Expression Pattern of ZDHHCs in KIRC

We first explored the expression pattern of ZDHHCs in KIRC using the TCGA database. As shown in **Figures 1A, B**, by evaluating the expression of ZDHHCs in 538 KIRC patient tissues and 72 normal tissues as well as 72 paired KIRC tissues and corresponding adjacent normal tissues, we found that ZDHHC2, 3, 6, 9, 14, 15, 16, 17, 18, 19, 20, 21, 22, and 23 had consistent and significant expression differences. Next, we used the GEPIA database to verify these ZDHHCs (**Figure 1C**). After further excluding ZDHHC16 and 22 that had no significant difference in expression, we finally determined the expression of ZDHHC2, 3, 6, 14, 15, 21, and 23 was significantly down-regulated whereas the expression of ZDHHC9, 17, 18, 19, and 20 was significantly up-regulated in KIRC patient tissues vs. normal tissues.

**Figure 2:**
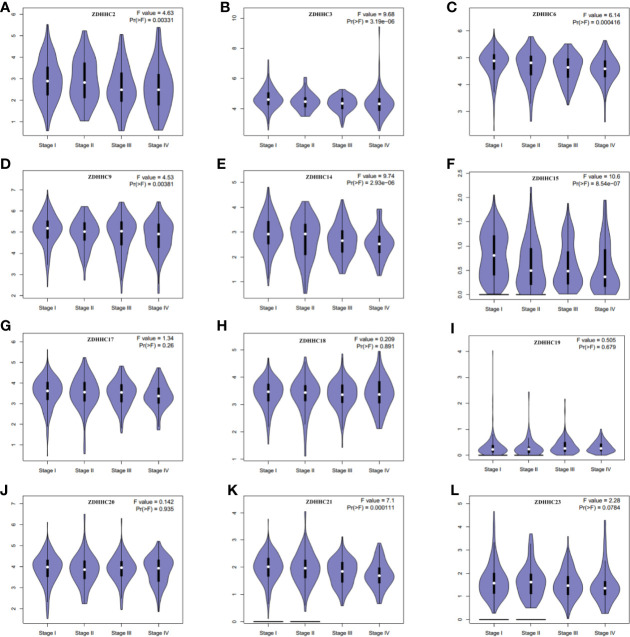
The expression patterns of these differentially expressed ZDHHCs in the main pathological stages of kidney renal clear cell carcinoma (KIRC) patients (GEPIA). The expression patterns of **(A)** ZDHHC2, **(B)** ZDHHC3, **(C)** ZDHHC6, **(D)** ZDHHC9, **(E)** ZDHHC14, **(F)** ZDHHC15, **(G)** ZDHHC17, **(H)** ZDHHC18, **(I)** ZDHHC19, **(J)** ZDHHC20, **(K)** ZDHHC21, **(L)** ZDHHC23 in the main pathological stage of KIRC patients. One-way ANOVA was used to determine gene expression difference among the pathological stage of KIRC patients and a value of P < 0.05 was considered statistically significant.

Then we further evaluated the expression patterns of these differentially expressed ZDHHCs in the main pathological stages of KIRC patients by generating expression violin plots using GEPIA (**Figures 2A–L**), and found a significant correlation between the expression of ZDHHC2, 3, 6, 9, 14, 15, 21 and the pathological stage of KIRC patients. With the progression of tumors, the expression of ZDHHC2, 3, 6, 9, 14, 15, 21 decreased significantly, indicating that these ZDHHCs played an important role in the progression and tumorigenesis of KIRC.

### Prognostic Value of Differentially Expressed ZDHHCs in KIRC

**Figure 3:**
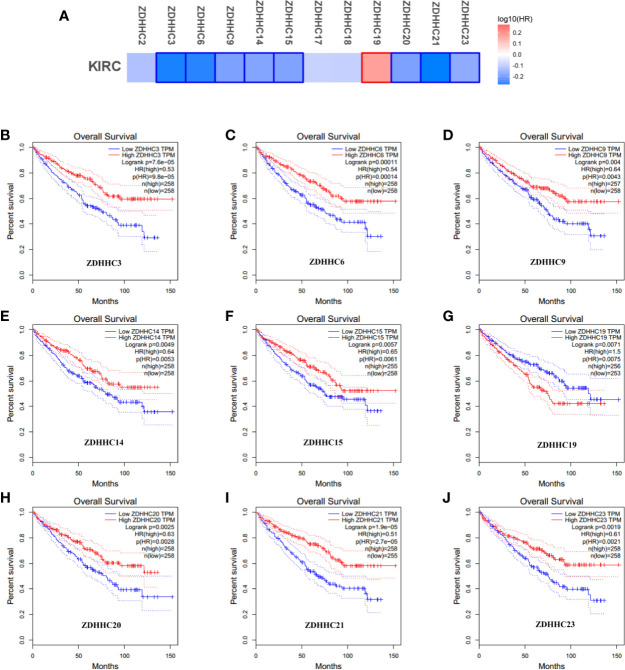
The effect of differentially expressed ZDHHCs on the overall survival of kidney renal clear cell carcinoma (KIRC) patients (GEPIA). **(A)** Survival significance map of differentially expressed ZDHHCs showed the over survival analysis results based on the cox proportional hazard ratio (HR) through GEPIA (the red and blue blocks denoted higher and lower risks, respectively; the rectangles with frames indicated significant unfavorable and favorable results). The overall survival curve of **(B)** ZDHHC3, **(C)** ZDHHC6, **(D)** ZDHHC9, **(E)** ZDHHC14, **(F)** ZDHHC15, **(G)** ZDHHC19, **(H)** ZDHHC20, **(I)** ZDHHC21, **(J)** ZDHHC23 in KIRC. The group cutoff choice for overall survival was the median. A log-rank test was used to estimate the difference in overall survival and a value of P < 0.05 was considered statistically significant.

**Figure 4:**
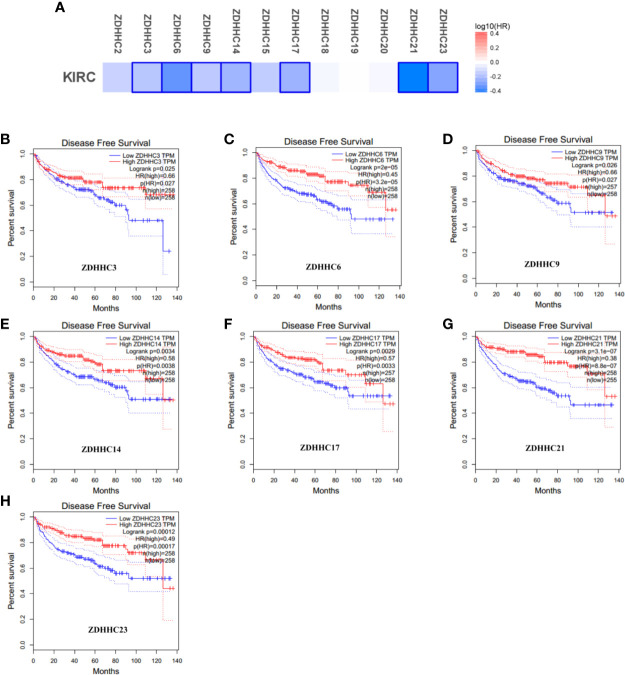
The effect of differentially expressed ZDHHCs on disease-free survival of kidney renal clear cell carcinoma (KIRC) patients (GEPIA). **(A)** Survival significance map of differentially expressed ZDHHCs showed the disease-free survival analysis results based on the cox proportional hazard ratio (HR) through GEPIA (blue blocks denoted higher and lower risks, respectively; the rectangles with frames indicated significant favorable results). The disease-free survival curve of **(B)** ZDHHC3, **(C)** ZDHHC6, **(D)** ZDHHC9, **(E)** ZDHHC14, **(F)** ZDHHC17, **(G)** ZDHHC21, **(H)** ZDHHC23 in KIRC. The group cutoff choice for disease-free survival was the median. A log-rank test was used to estimate the difference in disease-free survival and a value of P < 0.05 was considered statistically significant.

To explore the prognostic value of the ZDHHCs in patients with KIRC, we assessed the impact of differentially expressed ZDHHCs on clinical outcomes including overall survival and disease-free survival using GEPIA. The survival significance map (**Figures 3A** and **4A**) was drawn based on the cox proportional hazard ratio (HR). We found that high expression of ZDHHC3, 6, 9, 14, 15, 20, 21, 23 was significantly favorable whereas high expression of ZDHHC19 was significantly unfavorable for the overall survival of KIRC patients (**Figure 3A**). And high expression of ZDHHC3, 6, 9, 14, 17, 21, 23 was significantly favorable for the disease-free survival of KIRC patients (**Figure 4A**). The Kaplan-Meier plots of these ZDHHCs having significant impacts on the overall survival or disease-free survival of KIRC patients were further presented in **Figures 3** and **4**. We found that KIRC patients with decreased ZDHHC3, 6, 9, 14, 15, 20, 21, 23 expressions and increased ZDHHC19 expression were strongly associated with poor overall survival (**Figures 3B–J**). And KIRC patients with decreased ZDHHC3, 6, 9, 14, 17, 21, 23 expressions were significantly associated with poor disease-free survival (**Figures 4B–H**). In view of the prognostic value of ZDHHC3, 6, 9, 14, 15, 17, 19, 20, 21, and 23, we conducted the following analysis on these 10 differently expressed ZDHHCs.

### The Correlation of Immune Cell Infiltration With 10 Differentially Expressed ZDHHCs in KIRC Patient Tissues

**Figure 5:**
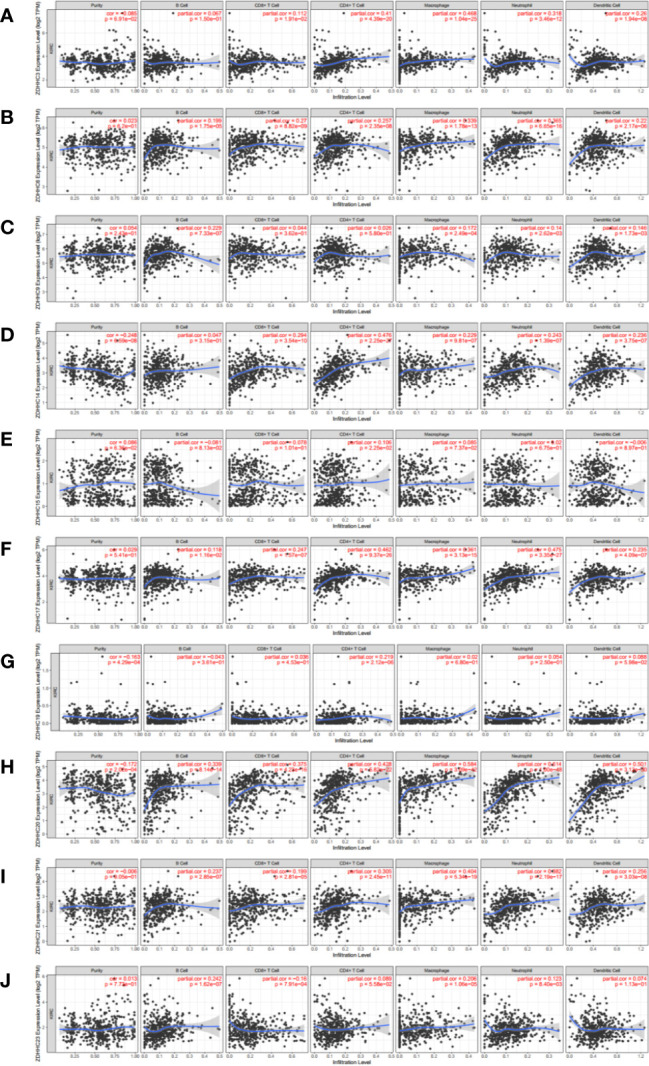
The correlation of immune cell infiltration with 10 differentially expressed ZDHHCs in kidney renal clear cell carcinoma (KIRC) patient tissues (Timer). The correlation between the abundance of immune cells and the expression of **(A)** ZDHHC3, **(B)** ZDHHC6, **(C)** ZDHHC9, **(D)** ZDHHC14, **(E)** ZDHHC15, **(F)** ZDHHC17, **(G)** ZDHHC19, **(H)** ZDHHC20, **(I)** ZDHHC21, **(J)** ZDHHC23 in KIRC. Spearman correlation coefficient was chosen for the correlation analysis and a value of P < 0.05 was considered statistically significant.

Immune cell infiltration is an important determinant of immune response and prognosis in cancer patients, including those with KIRC (21). Thus, we analyzed the correlation between the expression of these 10 differentially expressed ZDHHCs and infiltration of six immune cell types, including B cells, CD8+ T cells, CD4+ T cells, macrophages, neutrophils, and dendritic cells using the Time database (**Figures 5A–J**). Interestingly, the expression of ZDHHC6, 17, 20, 21 was significantly and positively correlated with the infiltration of all the six immune cell types and the expression of ZDHHC3 and 14 was significantly and positively correlated with the infiltration of five immune cell types excluding B cells. Further, ZDHHC9 expression was significantly and positively correlated with the infiltration of B cells, macrophages, neutrophils, and dendritic cells. ZDHHC23 expression was significantly and positively correlated with the infiltration of B cells, macrophages, and neutrophils and negatively correlated with the infiltration of CD8+ T cells. Besides, the expression of ZDHHC15 and 19 was significantly and positively correlated with the infiltration of CD4+ T cells.

### Gene Alterations, Expression Correlation, Micro(mi)RNA Networks, Co-Expression, and Interaction Analyses of 10 Differentially Expressed ZDHHCs in KIRC

**Figure 6:**
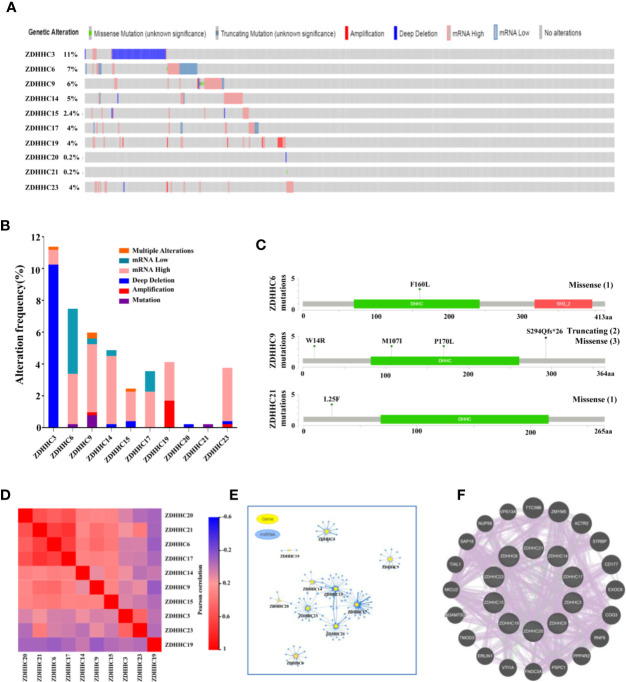
Gene alterations, expression correlation, micro(mi)RNA networks, co-expression and interaction analyses of 10 differentially expressed ZDHHCs in kidney renal clear cell carcinoma (KIRC). **(A, B)** Summary of gene alterations of differentially expressed ZDHHCs in KIRC (cBioPortal). **(C)** The mutations of ZDHHC6, 9, and 21 were plotted (cBioPortal) and (1), (2) and (3) represent the number of mutation sites. **(D)** Correlation heat map of different expressed ZDHHCs in KIRC (GEPIA). **(E)** miRNA network of different expressed ZDHHCs in KIRC (GSCALite). **(F)** Co-expression and interaction analyses of differentially expressed ZDHHCs in KIRC (GeneMANIA). The purple lines represent co-expression, and the green lines represent interaction.

We then performed a comprehensive analysis of the molecular characteristics of these 10 differentially expressed ZDHHCs in KIRC. The gene alterations for these ZDHHCs in KIRC was analyzed using cBioPortal. A total of 538 KIRC patients (TCGA, firehose legacy) were selected. The frequency of ZDHHC gene alterations in KIRC, mainly including multiple alterations, mRNA low, mRNA high, deep deletion, amplification, and mutation, varied from 0.2% to 11%, respectively (**Figure 6A**). High mRNA expression was the most common type of gene alteration in these samples and deep deletion was the major type of gene alteration of ZDHHC3 (**Figure 6B**). The missense mutation was identified in ZDHHC6, 9, and 21 (**Figure 6C**). The truncating mutation was found in ZDHHC9 (**Figure 6C**). We next explored the expression correlation among these ZDHHCs and found that there was a high correlation among the expression of ZDHHC6, 17, 20, and 21 and a low to moderate correlation among ZDHHC3, 9, 14, 15, 19, and 23 (**Figure 6D**, **Table S1**). We also analyzed the miRNA network involved with these ZDHHCs using GSCALite. As shown in **Figure 6E**, there were more miRNAs to potentially regulate ZDHHC3, 6, 9, 14, 15, 17, 21, and 23 than ZDHHC19 and 20 (**Figure 6E**, **Table S2**). A protein-protein interaction (PPI) network constructed using the String database indicated that there was little interaction among these ZDHHCs (**Figure S1A**). As expected, co-expression and interaction analyses (**Figure 6F**) from GeneMANIA revealed that the functions of these ZDHHCs were primarily related to protein palmitoylation, protein acylation, lipoprotein biosynthetic and metabolic process.

### Predicted Functions of 10 Differently Expressed ZDHHCs in KIRC

**Figure 7:**
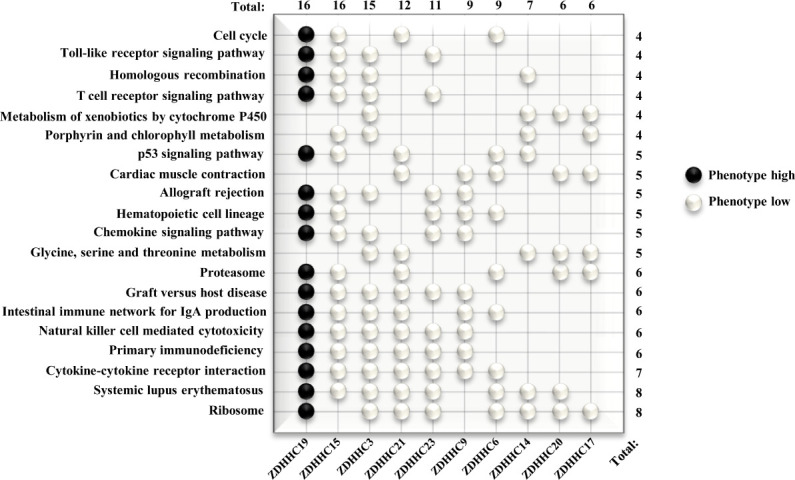
Predicted Functions of 10 differently expressed ZDHHCs in kidney renal clear cell carcinoma (KIRC) by GSEA. Pathway enrichment for differently expressed ZDHHCs in KIRC was shown in the WeiQi diagram. Black stones represent pathways enriched in phenotype high, and white stones represent pathways enriched in phenotype low. The number (right) represents the number of ZDHHCs associated with the enrichment of the pathway and the number (top) represents the number of pathways enriched by the gene. The V7.0.Gene set in the gene sets database and 1000 for the number of permutations were selected for each analysis and all gene sets were significantly enriched at nominal p < 5% and FDR < 25%.

We further investigated potential mechanisms of these 10 differently expressed ZDHHCs in KIRC by the GSEA method. Pathways with higher frequency enriched in phenotype high of ZDHHC19 and in phenotype low of ZDHHC3, 6, 14, 15, 17, 19, 20, 21, and 23 were shown in the WeiQi diagram (**Figure 7**), indicating that most of these ZDHHCs were closely related to immune-correlated signal pathways. ZDHHC19, 15, 3, 21, 23, 6, 14, and 20 were related to systemic lupus erythematosus. ZDHHC19, 15, 3, 21, 23, and 9 were involved in primary immunodeficiency and natural killer cell-mediated cytotoxicity. ZDHHC19, 15, 3, 21, 9, and 6 were connected with the intestinal immune network for IgA production. ZDHHC19, 15, 3, and 23 were associated with T cell receptor signaling pathway. Moreover, many of these ZDHHCs were closely linked to metabolism-related signaling pathways. ZDHHC19, 3, 21, 23, 6, 14, 20, and 17 were associated with ribosome pathway. ZDHHC3, 21, 14, 20, and 17 were connected with glycine, serine and threonine metabolism. ZDHHC15, 3, 14, and 17 were associated with porphyrin and chlorophyll metabolism. ZDHHC3, 14, 20, and 17 were related to metabolism of xenobiotics by cytochrome P450. The results of enrichment analysis for these 10 ZDHHCs were shown in **Table S3**, which also showed that oncogenic signaling activation was associated with certain ZDHHCs. For example, the JAK/STAT signaling pathway was enriched in phenotype high of ZDHHC19 and in phenotype low of ZDHHC15 and 23. The Wnt signaling pathway was activated by down-expressed ZDHHC6, 9, and 23. Taken together, these results suggested that the immune or metabolic disorders or the activation of oncogenic signaling pathways caused by abnormal expression of these ZDHHCs may be important mechanisms of tumor progression and poor prognosis in patients with KIRC.

## Discussion

The 23 human ZDHHC family members were firstly identified in 2004 (22). These ZDHHCs have homologous and highly conserved Asp-His-His-Cys (D-H-H-C) tetrapeptide motifs that are directly involved in the palmitoyl transfer reaction (23). However, because of the variable extent of palmitoyl acyltransferase activity and specificity of substrate proteins, all ZDHHCs play an indispensable role in multiple cellular biological processes and signaling pathways (24). Emerging evidence has indicated that ZDHHCs not only regulate normal physiological processes but are also involved in a variety of disease states including cancer (25). Over the past decade, numerous studies showed that these ZDHHCs modulated the function of important oncogenes and tumor suppressors and often displayed altered expression patterns in cancer (6, 26). Their abnormal expression and loss of function affect tumor progression, metastasis, the efficacy of cancer treatment, and patient prognosis (5). Thus, a better understanding of ZDHHCs in KIRC will be necessary for future development of ZDHHCs-based therapy.

In this study, we first evaluated the expression pattern of the 23 ZDHHCs in KIRC based on TCGA and GEPIA database. We found that 12 genes were differentially expressed in KIRC vs. normal tissues (ZDHHC2, 3, 6, 14, 15, 21, and 23 were down-regulated; ZDHHC9, 17, 18, 19, and 20 were up-regulated). We further evaluated the expression patterns of these differentially expressed ZDHHCs in the main pathological stages of KIRC patients and demonstrated that the expression of ZDHHC2, 3, 6, 9, 14, 15, 21 in tumors decreased with the increase of the pathological stage of KIRC patients. These data suggested that these differentially expressed ZDHHCs may play a significant role in the tumorigenesis and progression of KIRC.

To evaluate whether these differentially expressed ZDHHCs can be used as molecular markers to predict the survival of KIRC patients or to guide clinical treatment, we analyzed the impact of differentially expressed ZDHHCs on the prognosis of KIRC patients. Interestingly, we found that 10 of 12 differently expressed ZDHHCs were significantly associated with the prognosis of KIRC patients. In detail, KIRC patients with down-regulation of ZDHHC3, 6, 9, 14, 15, 20, 21, 23, and up-regulation of ZDHHC19 were strongly associated with poor overall survival and KIRC patients with down-regulation of ZDHHC3, 6, 9, 14, 17, 21, and 23 were significantly associated with poor disease-free survival. These results indicated the potential of ZDHHC3, 6, 9, 14, 15, 17, 19, 20, 21, 23 as prognostic markers or molecular targets.

Immune cells that infiltrate tumors form an ecosystem in the tumor microenvironment to regulate cancer progression and are closely associated with clinical outcome in KIRC (27). Tumor immune infiltrating cells mainly include innate immune cells (such as macrophages, neutrophils, and dendritic cells) and adaptive immune cells (T and B lymphocytes). These diverse immune cells communicate directly or indirectly with each other and together control the growth of tumor cells (28). Therefore, tumor-infiltrating immune cells are expected to be effective targets for improving clinical outcomes. In this study, we evaluated the correlation between 10 differentially expressed ZDHHCs and tumor infiltration of six immune cell types, including B cells, CD8+ T cells, CD4+ T cells, macrophages, neutrophils, and dendritic cells, and found a significant correlation between differentially expressed ZDHHCs and the infiltrating immune cells. These results revealed that these 10 ZDHHCs may regulate the immune status of KIRC patients, which may be an important factor affecting tumor progression and patient prognosis.

Tumorigenesis and progression of KIRC are complex and multifaceted, and genetic alterations also play an important role in this process (29). Thus, we evaluated the genetic alterations of these 10 differentially expressed ZDHHCs in KIRC and found that there were frequent genetic alterations in these ZDHHCs and high mRNA expression was the most common type of gene alteration. We also explored the potential expression correlation of these 10 ZDHHCs and found that there was a high correlation among the expression of ZDHHC6, 17, 20, and 21. As expected, co-expression and interaction network analysis showed that the functions of these ZDHHCs were mainly protein palmitoylation and acetylation, etc. In addition, in the past few decades, miRNAs have been shown to affect tumor progression and patient prognosis by inhibiting transcription or degrading mRNAs of target protein (30, 31). The miRNA network indicated that these differentially expressed ZDHHCs were potentially regulated by miRNA.

We then focused on the potential mechanisms of action of these 10 differentially expressed ZDHHCs by GSEA. Consistent with the results of immune cell infiltration (**Figures 5A–L**), most of these ZDHHCs were closely related to immune-correlated signal pathways such as systemic lupus erythematosus, immunodeficiency and natural killer cell mediated cytotoxicity, suggesting that these ZDHHCs may play important regulatory roles in the immune microenvironment of KIRC. Historically, KIRC is one of the few tumors for which immunotherapy is effective (32). And the development and application of immune-related targeted therapy agents have been proven to be feasible and effective for the treatment of KIRC patients (33). However, the genes for the development of targeted approaches to KIRC immunotherapy have not been well identified. In view of the important roles of differentially expressed ZDHHCs on the KIRC immune environment, the development of immunomodulatory therapy targeting ZDHHCs may bring survival benefits to KIRC patients. Previous research has also shown that KIRC is closely related to reprogramming in cellular metabolism and is therefore also described as a **“**metabolic disease**”** (34, 35). Changes in metabolic gene expression patterns and abnormalities in metabolic-related pathways such as protein or amino acid metabolism are considered important causes of KIRC metabolic reprogramming, seriously affecting the prognosis of KIRC patients. Thus, targeting metabolic reprogramming in KIRC will also be important in future therapeutic planning. In this study, GSEA analyses also showed that many of these 10 differentially expressed ZDHHCs were closely related to abnormalities in metabolic pathways such as ribosome, amino acid metabolism, porphyrin and chlorophyll metabolism and metabolism of xenobiotics by cytochrome P450, suggesting the important functions of ZDHHCs in cellular metabolism. Besides, we also found that many well-known oncogenic signaling activations including JAK/STAT signaling and Wnt signaling were associated with abnormal expression of certain ZDHHCs. Taken together, the analysis above suggested that these ZDHHCs had potential as therapeutic targets in the clinical treatment of KIRC.

In conclusion, we performed a systematic analysis of ZDHHC expression patterns, prognostic value, immune infiltration, molecular characteristics and signaling pathways involved in KIRC. We hope our results will provide novel insight for identifying tumor markers or molecular targets for the treatment of KIRC. However, further research including *in vivo* and *in vitro* experiments is needed to validate our findings and promote further understanding of the ZDHHCs in KIRC.

## Data Availability Statement

Publicly available datasets were analyzed in this study. RNA-Seq data was downloaded from the TCGA database (https://portal.gdc.cancer.gov/) and the data of genetic alterations were obtained from cBioPortal (http://www.cbioportal.org/study/summary?id=kirc_tcga).

## Author Contributions

ZL and BX developed the idea and designed the research. ZL and MX analyzed the data. ZL and CL drafted the manuscript. YH, YZ, and BX revised the writing. All authors contributed to the article and approved the submitted version.

## Funding

This work was supported by grants from the National Natural Science Foundation of China [81672743 and 81974464], Shenzhen Basic Research Project (JCYJ20160331114230843), Tianjin Medical University Cancer Institute and Hospital Innovation Fund [1803], and Beijing Tianjin Hebei Basic Research Cooperation Project (Grant No. 19JCZDJC64500(Z)).

## Conflict of Interest

The authors declare that the research was conducted in the absence of any commercial or financial relationships that could be construed as a potential conflict of interest.
