# Supplementary material for: Bioinformatics Analysis of the Prognostic and Biological Significance of ZDHHC-Protein Acyltransferases in Kidney Renal Clear Cell Carcinoma
Source: Front Oncol. 2020 Dec 8;10:565414. doi: 10.3389/fonc.2020.565414 (PMC7753182; doi:10.3389/fonc.2020.565414)
Supplement: Supplementary file 1 [file DataSheet_1.zip › Supplementary files/Table S2.docx]

| Table S2. miRNAs that potentially regulated ten differentially expressed ZDHHCs(GSCALite). | |
| --- | --- |
| ZDHHCs | Name of miRNAs |
| ZDHHC3 | miR-320d |
|  | miR-181c-5p |
|  | miR-320b |
|  | miR-3147 |
|  | miR-320c |
|  | miR-181b-5p |
|  | miR-7-5p |
|  | miR-543 |
|  | miR-181a-5p |
|  | miR-320a |
|  | miR-98-5p |
| ZDHHC6 | miR-423-5p |
|  | miR-525-5p |
|  | miR-3133 |
|  | miR-154-5p |
|  | miR-890 |
|  | miR-26a-5p |
|  | miR-3184-5p |
|  | miR-136-5p |
|  | miR-520a-5p |
|  | miR-135a-5p |
| ZDHHC9 | miR-20b-5p |
|  | miR-199b-5p |
|  | miR-765 |
|  | miR-520h |
|  | miR-106a-5p |
|  | miR-199a-5p |
|  | miR-361-5p |
|  | miR-93-5p |
|  | miR-660-5p |
|  | miR-1262 |
| ZDHHC14 | miR-519c-3p |
|  | miR-519b-3p |
|  | miR-519a-3p |
|  | miR-2110 |
|  | miR-16-5p |
|  | miR-15b-5p |
|  | miR-15a-5p |
|  | miR-424-5p |
| ZDHHC15 | miR-2110 |
|  | miR-16-5p |
|  | miR-15b-5p |
|  | miR-15a-5p |
|  | miR-590-3p |
|  | miR-582-5p |
|  | miR-3163 |
|  | miR-877-5p |
|  | miR-548u |
|  | miR-145-5p |
|  | miR-190b |
|  | miR-3164 |
|  | miR-196b-5p |
|  | miR-196a-5p |
|  | miR-1284 |
|  | miR-198 |
|  | miR-625-5p |
|  | miR-629-5p |
|  | miR-449a |
|  | miR-206 |
|  | miR-142-5p |
|  | miR-34a-5p |
|  | miR-3125 |
|  | miR-548n |
|  | miR-3121-3p |
|  | miR-556-3p |
|  | miR-148b-3p |
| ZDHHC17 | miR-148a-3p |
|  | miR-148b-3p |
|  | miR-3173-3p |
|  | miR-3161 |
|  | miR-5480-3p |
|  | miR-429 |
|  | miR-629-5p |
|  | miR-200b-3p |
|  | miR-34a-5p |
|  | miR-548d-3p |
|  | miR-1976 |
|  | miR-944 |
|  | miR-27b-3p |
|  | miR-1236-3p |
|  | miR-211-5p |
|  | miR-34c-3p |
|  | miR-200c-3p |
|  | miR-548k |
|  | miR-548u |
|  | miR-548b-3p |
|  | miR-548t-5p |
|  | miR-576-5p |
|  | miR-30b-5p |
|  | miR-3164 |
|  | miR-3158-3p |
|  | miR-3163 |
|  | miR-27a-3p |
| ZDHHC19 | miR-24-3p |
|  | miR-31-5p |
| ZDHHC20 | miR-335-5p |
|  | miR-873-5p |
|  | miR-516b-5p |
|  | miR-578 |
| ZDHHC21 | miR-548t-5p |
|  | miR-576-5p |
|  | miR-30b-5p |
|  | miR-548a-5p |
|  | miR-548l |
|  | miR-590-3p |
|  | miR-337-3p |
|  | miR-513a-3p |
|  | miR-548w |
|  | miR-1290 |
|  | miR-483-3p |
|  | miR-30e-5p |
|  | miR-3165 |
|  | miR-30c-5p |
|  | miR-548s |
|  | miR-452-5p |
|  | miR-643 |
|  | miR-30d-5p |
|  | miR-196b-5p |
|  | miR-548x-3p |
|  | miR-548b-5p |
| ZDHHC23 | miR-424-5p |
|  | miR-15a-5p |
|  | miR-195-5p |
|  | miR-873-5p |
|  | miR-129-5p |
|  | miR-143-3p |
|  | miR-130-3p |
|  | miR-337-3p |
|  | miR-1179 |
|  | miR-513c-5p |
|  | miR-301a-3p |
|  | miR-497-5p |
|  | miR-3161 |
|  | miR-454-3p |
|  | miR-513a-3p |
|  | miR-1185-5p |
|  | miR-130a-3p |
|  | miR-409-3p |
|  | miR-3142 |
|  | miR-513a-5p |
