# Supplementary material for: Bioinformatics Analysis of the Prognostic and Biological Significance of ZDHHC-Protein Acyltransferases in Kidney Renal Clear Cell Carcinoma
Source: Front Oncol. 2020 Dec 8;10:565414. doi: 10.3389/fonc.2020.565414 (PMC7753182; doi:10.3389/fonc.2020.565414)
Supplement: Supplementary file 1 [file DataSheet_1.zip › Supplementary files/Table S3.docx]

| **Table S3. The results of enrichment analysis for ten differentially expressed ZDHHCs.** | | | | |
| --- | --- | --- | --- | --- |
| ZDHHCs | Pathway names | NES | NOM p-val | FDR q-val |
| ZDHHC3 | KEGG_PENTOSE_AND_GLUCURONATE_INTERCONVERSIONS | -2.41426 | 0 | 0 |
|  | KEGG_DRUG_METABOLISM_OTHER_ENZYMES | -2.3341 | 0 | 0 |
|  | KEGG_STARCH_AND_SUCROSE_METABOLISM | -2.3321 | 0 | 0 |
|  | KEGG_ASCORBATE_AND_ALDARATE_METABOLISM | -2.23584 | 0 | 0 |
|  | KEGG_ALLOGRAFT_REJECTION | -2.20687 | 0 | 3.71E-04 |
|  | KEGG_GRAFT_VERSUS_HOST_DISEASE | -2.19653 | 0 | 3.09E-04 |
|  | KEGG_SYSTEMIC_LUPUS_ERYTHEMATOSUS | -2.17864 | 0 | 2.65E-04 |
|  | KEGG_PORPHYRIN_AND_CHLOROPHYLL_METABOLISM | -2.12573 | 0 | 2.32E-04 |
|  | KEGG_AUTOIMMUNE_THYROID_DISEASE | -2.06558 | 0 | 3.58E-04 |
|  | KEGG_ANTIGEN_PROCESSING_AND_PRESENTATION | -2.06083 | 0 | 3.22E-04 |
|  | KEGG_INTESTINAL_IMMUNE_NETWORK_FOR_IGA_PRODUCTION | -1.96667 | 0 | 0.001684 |
|  | KEGG_PRIMARY_IMMUNODEFICIENCY | -1.96012 | 0 | 0.001678 |
|  | KEGG_STEROID_HORMONE_BIOSYNTHESIS | -1.92891 | 0 | 0.001914 |
|  | KEGG_HISTIDINE_METABOLISM | -1.91961 | 0 | 0.002017 |
|  | KEGG_DRUG_METABOLISM_CYTOCHROME_P450 | -1.90547 | 0 | 0.002091 |
|  | KEGG_RETINOL_METABOLISM | -1.88708 | 0 | 0.002486 |
|  | KEGG_TYPE_I_DIABETES_MELLITUS | -1.86153 | 0.002809 | 0.003059 |
|  | KEGG_METABOLISM_OF_XENOBIOTICS_BY_CYTOCHROME_P450 | -1.8193 | 0 | 0.005378 |
|  | KEGG_GLYCINE_SERINE_AND_THREONINE_METABOLISM | -1.8186 | 0 | 0.005189 |
|  | KEGG_PPAR_SIGNALING_PATHWAY | -1.73816 | 0 | 0.012212 |
|  | KEGG_PEROXISOME | -1.73613 | 0.003953 | 0.012098 |
|  | KEGG_ALANINE_ASPARTATE_AND_GLUTAMATE_METABOLISM | -1.70436 | 0.005634 | 0.015496 |
|  | KEGG_CYTOKINE_CYTOKINE_RECEPTOR_INTERACTION | -1.67331 | 0 | 0.020355 |
|  | KEGG_RENIN_ANGIOTENSIN_SYSTEM | -1.66591 | 0.037037 | 0.020394 |
|  | KEGG_FRUCTOSE_AND_MANNOSE_METABOLISM | -1.65394 | 0.002857 | 0.021159 |
|  | KEGG_TRYPTOPHAN_METABOLISM | -1.5946 | 0.009146 | 0.033817 |
|  | KEGG_HOMOLOGOUS_RECOMBINATION | -1.58769 | 0.017903 | 0.034926 |
|  | KEGG_CYTOSOLIC_DNA_SENSING_PATHWAY | -1.57982 | 0.006231 | 0.03597 |
|  | KEGG_NATURAL_KILLER_CELL_MEDIATED_CYTOTOXICITY | -1.5467 | 0 | 0.044805 |
|  | KEGG_BETA_ALANINE_METABOLISM | -1.52012 | 0.031339 | 0.053426 |
|  | KEGG_ASTHMA | -1.49012 | 0.034268 | 0.064441 |
|  | KEGG_T_CELL_RECEPTOR_SIGNALING_PATHWAY | -1.44603 | 0 | 0.087152 |
|  | KEGG_MATURITY_ONSET_DIABETES_OF_THE_YOUNG | -1.44208 | 0.039788 | 0.087197 |
|  | KEGG_MISMATCH_REPAIR | -1.43627 | 0.048257 | 0.085236 |
|  | KEGG_TOLL_LIKE_RECEPTOR_SIGNALING_PATHWAY | -1.40176 | 0.015326 | 0.10615 |
|  | KEGG_RIBOSOME | -1.32881 | 0.019305 | 0.163407 |
|  | KEGG_CHEMOKINE_SIGNALING_PATHWAY | -1.26415 | 0.027473 | 0.214469 |
| ZDHHC6 | KEGG_BASAL_CELL_CARCINOMA | -1.83705 | 0 | 0.039093 |
|  | KEGG_RIBOSOME | -1.75794 | 0 | 0.048258 |
|  | KEGG_ECM_RECEPTOR_INTERACTION | -1.731 | 0 | 0.046029 |
|  | KEGG_HEDGEHOG_SIGNALING_PATHWAY | -1.68185 | 0.004155 | 0.071632 |
|  | KEGG_ALPHA_LINOLENIC_ACID_METABOLISM | -1.66435 | 0.00317 | 0.067823 |
|  | KEGG_ARRHYTHMOGENIC_RIGHT_VENTRICULAR_CARDIOMYOPATHY_ARVC | -1.6355 | 0 | 0.080061 |
|  | KEGG_PRION_DISEASES | -1.60823 | 0.007299 | 0.094803 |
|  | KEGG_P53_SIGNALING_PATHWAY | -1.59214 | 0.005348 | 0.099336 |
|  | KEGG_HYPERTROPHIC_CARDIOMYOPATHY_HCM | -1.5867 | 0.002594 | 0.093976 |
|  | KEGG_COMPLEMENT_AND_COAGULATION_CASCADES | -1.57625 | 0.005405 | 0.096014 |
|  | KEGG_GLYCOSAMINOGLYCAN_BIOSYNTHESIS_CHONDROITIN_SULFATE | -1.56555 | 0.021739 | 0.096598 |
|  | KEGG_WNT_SIGNALING_PATHWAY | -1.55835 | 0.002361 | 0.095339 |
|  | KEGG_AXON_GUIDANCE | -1.53161 | 0.003769 | 0.114503 |
|  | KEGG_FOCAL_ADHESION | -1.51773 | 0.001149 | 0.12113 |
|  | KEGG_ETHER_LIPID_METABOLISM | -1.5155 | 0.019259 | 0.115835 |
|  | KEGG_CALCIUM_SIGNALING_PATHWAY | -1.50998 | 0 | 0.115112 |
|  | KEGG_MELANOGENESIS | -1.50387 | 0.007643 | 0.114791 |
|  | KEGG_PROTEASOME | -1.4861 | 0.024828 | 0.129581 |
|  | KEGG_HEMATOPOIETIC_CELL_LINEAGE | -1.48049 | 0.009459 | 0.129708 |
|  | KEGG_INTESTINAL_IMMUNE_NETWORK_FOR_IGA_PRODUCTION | -1.47888 | 0.022222 | 0.125048 |
|  | KEGG_SYSTEMIC_LUPUS_ERYTHEMATOSUS | -1.47272 | 0.006105 | 0.125504 |
|  | KEGG_CYTOKINE_CYTOKINE_RECEPTOR_INTERACTION | -1.47216 | 0.002262 | 0.120312 |
|  | KEGG_PATHOGENIC_ESCHERICHIA_COLI_INFECTION | -1.45787 | 0.036671 | 0.131287 |
|  | KEGG_CARDIAC_MUSCLE_CONTRACTION | -1.45094 | 0.023873 | 0.134308 |
|  | KEGG_LONG_TERM_DEPRESSION | -1.41851 | 0.02815 | 0.170062 |
|  | KEGG_NEUROACTIVE_LIGAND_RECEPTOR_INTERACTION | -1.41384 | 0.00339 | 0.170184 |
|  | KEGG_DILATED_CARDIOMYOPATHY | -1.39976 | 0.02584 | 0.184688 |
|  | KEGG_CELL_CYCLE | -1.39487 | 0.015326 | 0.185227 |
|  | KEGG_PATHWAYS_IN_CANCER | -1.39205 | 0.004329 | 0.182497 |
|  | KEGG_REGULATION_OF_ACTIN_CYTOSKELETON | -1.39187 | 0.005714 | 0.176612 |
|  | KEGG_TGF_BETA_SIGNALING_PATHWAY | -1.38182 | 0.034076 | 0.184306 |
| ZDHHC9 | KEGG_BASAL_CELL_CARCINOMA | -1.77921 | 0 | 0.005671 |
|  | KEGG_HEDGEHOG_SIGNALING_PATHWAY | -1.7635 | 0 | 0.004738 |
|  | KEGG_DILATED_CARDIOMYOPATHY | -1.67713 | 0 | 0.021744 |
|  | KEGG_HYPERTROPHIC_CARDIOMYOPATHY_HCM | -1.6636 | 0 | 0.020289 |
|  | KEGG_WNT_SIGNALING_PATHWAY | -1.61855 | 0 | 0.032246 |
|  | KEGG_CARDIAC_MUSCLE_CONTRACTION | -1.58005 | 0.003122 | 0.050005 |
|  | KEGG_TASTE_TRANSDUCTION | -1.57628 | 0.002101 | 0.045032 |
|  | KEGG_GRAFT_VERSUS_HOST_DISEASE | -1.54509 | 0.012277 | 0.064954 |
|  | KEGG_MELANOGENESIS | -1.54105 | 0.001019 | 0.060336 |
|  | KEGG_ECM_RECEPTOR_INTERACTION | -1.51158 | 0.003083 | 0.083897 |
|  | KEGG_PRIMARY_IMMUNODEFICIENCY | -1.48974 | 0.023569 | 0.101092 |
|  | KEGG_ARRHYTHMOGENIC_RIGHT_VENTRICULAR_CARDIOMYOPATHY_ARVC | -1.47819 | 0.010417 | 0.10626 |
|  | KEGG_CYTOKINE_CYTOKINE_RECEPTOR_INTERACTION | -1.46105 | 0 | 0.118576 |
|  | KEGG_TGF_BETA_SIGNALING_PATHWAY | -1.4478 | 0.006205 | 0.128369 |
|  | KEGG_HEMATOPOIETIC_CELL_LINEAGE | -1.44641 | 0.00925 | 0.121664 |
|  | KEGG_NATURAL_KILLER_CELL_MEDIATED_CYTOTOXICITY | -1.4448 | 0.002018 | 0.115843 |
|  | KEGG_INTESTINAL_IMMUNE_NETWORK_FOR_IGA_PRODUCTION | -1.41815 | 0.026172 | 0.145997 |
|  | KEGG_ALLOGRAFT_REJECTION | -1.40956 | 0.03876 | 0.151813 |
|  | KEGG_CALCIUM_SIGNALING_PATHWAY | -1.39529 | 0.003021 | 0.167076 |
|  | KEGG_CHEMOKINE_SIGNALING_PATHWAY | -1.36189 | 0.005025 | 0.205294 |
|  | KEGG_FOCAL_ADHESION | -1.35325 | 0.003003 | 0.204556 |
| ZDHHC14 | KEGG_RIBOSOME | -2.53243 | 0 | 0 |
|  | KEGG_PORPHYRIN_AND_CHLOROPHYLL_METABOLISM | -2.44997 | 0 | 0 |
|  | KEGG_ASCORBATE_AND_ALDARATE_METABOLISM | -2.3353 | 0 | 0 |
|  | KEGG_VALINE_LEUCINE_AND_ISOLEUCINE_DEGRADATION | -2.33434 | 0 | 0 |
|  | KEGG_CITRATE_CYCLE_TCA_CYCLE | -2.2797 | 0 | 0 |
|  | KEGG_PENTOSE_AND_GLUCURONATE_INTERCONVERSIONS | -2.22954 | 0 | 0 |
|  | KEGG_AMINO_SUGAR_AND_NUCLEOTIDE_SUGAR_METABOLISM | -2.15138 | 0 | 0.002245 |
|  | KEGG_DRUG_METABOLISM_OTHER_ENZYMES | -2.14717 | 0 | 0.001964 |
|  | KEGG_AMINOACYL_TRNA_BIOSYNTHESIS | -2.12736 | 0 | 0.001746 |
|  | KEGG_MISMATCH_REPAIR | -2.04602 | 0 | 0.002671 |
|  | KEGG_PROTEIN_EXPORT | -1.99258 | 0 | 0.003719 |
|  | KEGG_STEROID_HORMONE_BIOSYNTHESIS | -1.98066 | 0 | 0.003409 |
|  | KEGG_PYRUVATE_METABOLISM | -1.92708 | 0 | 0.00433 |
|  | KEGG_PROPANOATE_METABOLISM | -1.8656 | 0 | 0.007254 |
|  | KEGG_ALANINE_ASPARTATE_AND_GLUTAMATE_METABOLISM | -1.84603 | 0 | 0.008431 |
|  | KEGG_BUTANOATE_METABOLISM | -1.83893 | 0 | 0.009194 |
|  | KEGG_SYSTEMIC_LUPUS_ERYTHEMATOSUS | -1.8279 | 0 | 0.009488 |
|  | KEGG_DNA_REPLICATION | -1.77928 | 0 | 0.016086 |
|  | KEGG_HOMOLOGOUS_RECOMBINATION | -1.76164 | 0 | 0.016231 |
|  | KEGG_CYSTEINE_AND_METHIONINE_METABOLISM | -1.71852 | 0 | 0.020655 |
|  | KEGG_OXIDATIVE_PHOSPHORYLATION | -1.67686 | 0 | 0.027208 |
|  | KEGG_RETINOL_METABOLISM | -1.66538 | 0 | 0.028564 |
|  | KEGG_FRUCTOSE_AND_MANNOSE_METABOLISM | -1.65308 | 0.01087 | 0.0295 |
|  | KEGG_LINOLEIC_ACID_METABOLISM | -1.64169 | 0.015625 | 0.030744 |
|  | KEGG_NUCLEOTIDE_EXCISION_REPAIR | -1.60344 | 0 | 0.037576 |
|  | KEGG_PEROXISOME | -1.56904 | 0 | 0.045708 |
|  | KEGG_STARCH_AND_SUCROSE_METABOLISM | -1.4891 | 0 | 0.073256 |
|  | KEGG_ETHER_LIPID_METABOLISM | -1.47472 | 0.046296 | 0.077111 |
|  | KEGG_PENTOSE_PHOSPHATE_PATHWAY | -1.46554 | 0.034483 | 0.07903 |
|  | KEGG_PARKINSONS_DISEASE | -1.44665 | 0 | 0.084707 |
|  | KEGG_GLYCINE_SERINE_AND_THREONINE_METABOLISM | -1.43617 | 0.040323 | 0.088384 |
|  | KEGG_METABOLISM_OF_XENOBIOTICS_BY_CYTOCHROME_P450 | -1.39861 | 0.025 | 0.101719 |
|  | KEGG_DRUG_METABOLISM_CYTOCHROME_P450 | -1.37864 | 0 | 0.109065 |
|  | KEGG_P53_SIGNALING_PATHWAY | -1.36232 | 0.02439 | 0.116883 |
|  | KEGG_PYRIMIDINE_METABOLISM | -1.24432 | 0 | 0.185333 |
| ZDHHC15 | KEGG_SYSTEMIC_LUPUS_ERYTHEMATOSUS | -2.4116 | 0 | 0 |
|  | KEGG_CELL_CYCLE | -2.1943 | 0 | 0 |
|  | KEGG_HOMOLOGOUS_RECOMBINATION | -2.06282 | 0 | 0 |
|  | KEGG_GRAFT_VERSUS_HOST_DISEASE | -2.04042 | 0 | 0 |
|  | KEGG_CYTOKINE_CYTOKINE_RECEPTOR_INTERACTION | -2.03485 | 0 | 0 |
|  | KEGG_ALLOGRAFT_REJECTION | -2.03025 | 0 | 1.51E-04 |
|  | KEGG_INTESTINAL_IMMUNE_NETWORK_FOR_IGA_PRODUCTION | -2.01612 | 0 | 1.29E-04 |
|  | KEGG_PRIMARY_IMMUNODEFICIENCY | -1.96758 | 0.001689 | 7.25E-04 |
|  | KEGG_TYPE_I_DIABETES_MELLITUS | -1.90309 | 0 | 0.002263 |
|  | KEGG_P53_SIGNALING_PATHWAY | -1.83858 | 0 | 0.00572 |
|  | KEGG_T_CELL_RECEPTOR_SIGNALING_PATHWAY | -1.81734 | 0 | 0.006806 |
|  | KEGG_HEMATOPOIETIC_CELL_LINEAGE | -1.77008 | 0 | 0.010387 |
|  | KEGG_LINOLEIC_ACID_METABOLISM | -1.7209 | 0.001761 | 0.017112 |
|  | KEGG_MATURITY_ONSET_DIABETES_OF_THE_YOUNG | -1.7174 | 0.005199 | 0.016509 |
|  | KEGG_PROTEASOME | -1.67638 | 0.006908 | 0.025183 |
|  | KEGG_DNA_REPLICATION | -1.6502 | 0.00846 | 0.030984 |
|  | KEGG_LEISHMANIA_INFECTION | -1.64973 | 0.003185 | 0.029286 |
|  | KEGG_DRUG_METABOLISM_OTHER_ENZYMES | -1.62596 | 0.011327 | 0.036133 |
|  | KEGG_ANTIGEN_PROCESSING_AND_PRESENTATION | -1.59654 | 0.006421 | 0.046686 |
|  | KEGG_NOD_LIKE_RECEPTOR_SIGNALING_PATHWAY | -1.59564 | 0.004918 | 0.044653 |
|  | KEGG_NATURAL_KILLER_CELL_MEDIATED_CYTOTOXICITY | -1.58508 | 0 | 0.047577 |
|  | KEGG_OOCYTE_MEIOSIS | -1.58292 | 0.00471 | 0.046124 |
|  | KEGG_PORPHYRIN_AND_CHLOROPHYLL_METABOLISM | -1.54011 | 0.018092 | 0.064706 |
|  | KEGG_COMPLEMENT_AND_COAGULATION_CASCADES | -1.5316 | 0.007924 | 0.066552 |
|  | KEGG_AUTOIMMUNE_THYROID_DISEASE | -1.51733 | 0.019934 | 0.071758 |
|  | KEGG_ASTHMA | -1.49859 | 0.027027 | 0.080659 |
|  | KEGG_ASCORBATE_AND_ALDARATE_METABOLISM | -1.48583 | 0.044068 | 0.083762 |
|  | KEGG_ARACHIDONIC_ACID_METABOLISM | -1.47289 | 0.0304 | 0.090292 |
|  | KEGG_AMINO_SUGAR_AND_NUCLEOTIDE_SUGAR_METABOLISM | -1.4611 | 0.036522 | 0.095838 |
|  | KEGG_CHEMOKINE_SIGNALING_PATHWAY | -1.45401 | 0.001443 | 0.098847 |
|  | KEGG_TOLL_LIKE_RECEPTOR_SIGNALING_PATHWAY | -1.42996 | 0.015576 | 0.11579 |
|  | KEGG_CYTOSOLIC_DNA_SENSING_PATHWAY | -1.39082 | 0.049404 | 0.149458 |
|  | KEGG_B_CELL_RECEPTOR_SIGNALING_PATHWAY | -1.38658 | 0.030255 | 0.146392 |
|  | KEGG_JAK_STAT_SIGNALING_PATHWAY | -1.37544 | 0.018786 | 0.143567 |
| ZDHHC17 | KEGG_OXIDATIVE_PHOSPHORYLATION | -3.4032595 | 0 | 0 |
|  | KEGG_RIBOSOME | -2.8965888 | 0 | 0 |
|  | KEGG_PARKINSONS_DISEASE | -2.797687 | 0 | 0 |
|  | KEGG_PROTEASOME | -2.7268035 | 0 | 0 |
|  | KEGG_LYSOSOME | -2.430029 | 0 | 0 |
|  | KEGG_CITRATE_CYCLE_TCA_CYCLE | -2.3934937 | 0 | 0 |
|  | KEGG_AMINO_SUGAR_AND_NUCLEOTIDE_SUGAR_METABOLISM | -2.3054688 | 0 | 0 |
|  | KEGG_VALINE_LEUCINE_AND_ISOLEUCINE_DEGRADATION | -2.217529 | 0 | 0 |
|  | KEGG_GLUTATHIONE_METABOLISM | -2.2115712 | 0 | 0 |
|  | KEGG_HUNTINGTONS_DISEASE | -2.1380064 | 0 | 0.001947121 |
|  | KEGG_PEROXISOME | -2.0011208 | 0 | 0.004017883 |
|  | KEGG_VIBRIO_CHOLERAE_INFECTION | -1.9678679 | 0 | 0.005433408 |
|  | KEGG_ALZHEIMERS_DISEASE | -1.9552355 | 0 | 0.005015453 |
|  | KEGG_COMPLEMENT_AND_COAGULATION_CASCADES | -1.8861792 | 0 | 0.006443662 |
|  | KEGG_AMINOACYL_TRNA_BIOSYNTHESIS | -1.8623075 | 0 | 0.008073151 |
|  | KEGG_ARGININE_AND_PROLINE_METABOLISM | -1.8309137 | 0 | 0.010350355 |
|  | KEGG_PYRIMIDINE_METABOLISM | -1.7758799 | 0 | 0.015596566 |
|  | KEGG_ALANINE_ASPARTATE_AND_GLUTAMATE_METABOLISM | -1.6904166 | 0 | 0.031334437 |
|  | KEGG_PHENYLALANINE_METABOLISM | -1.678142 | 0.015706806 | 0.03087523 |
|  | KEGG_GLYCINE_SERINE_AND_THREONINE_METABOLISM | -1.6395837 | 0.015748031 | 0.037926756 |
|  | KEGG_TYROSINE_METABOLISM | -1.6355244 | 0.024390243 | 0.03807912 |
|  | KEGG_BUTANOATE_METABOLISM | -1.6173352 | 0 | 0.040084068 |
|  | KEGG_N_GLYCAN_BIOSYNTHESIS | -1.6118419 | 0.01369863 | 0.039459474 |
|  | KEGG_PATHOGENIC_ESCHERICHIA_COLI_INFECTION | -1.5861492 | 0.016949153 | 0.046098806 |
|  | KEGG_FRUCTOSE_AND_MANNOSE_METABOLISM | -1.5684414 | 0 | 0.049815323 |
|  | KEGG_RNA_POLYMERASE | -1.5660546 | 0.014705882 | 0.048465103 |
|  | KEGG_TERPENOID_BACKBONE_BIOSYNTHESIS | -1.561172 | 0.045045044 | 0.048844185 |
|  | KEGG_GLYOXYLATE_AND_DICARBOXYLATE_METABOLISM | -1.513944 | 0.04519774 | 0.06274062 |
|  | KEGG_PYRUVATE_METABOLISM | -1.4750607 | 0.030927835 | 0.07621918 |
|  | KEGG_PRION_DISEASES | -1.4638505 | 0.029411765 | 0.082029454 |
|  | KEGG_PORPHYRIN_AND_CHLOROPHYLL_METABOLISM | -1.4167867 | 0.019417476 | 0.10525945 |
|  | KEGG_METABOLISM_OF_XENOBIOTICS_BY_CYTOCHROME_P450 | -1.3542895 | 0.02173913 | 0.13221943 |
|  | KEGG_CARDIAC_MUSCLE_CONTRACTION | -1.3279666 | 0 | 0.148362 |
| ZDHHC19 | KEGG_GRAFT_VERSUS_HOST_DISEASE | 2.279578 | 0 | 0 |
|  | KEGG_INTESTINAL_IMMUNE_NETWORK_FOR_IGA_PRODUCTION | 2.267721 | 0 | 0 |
|  | KEGG_ALLOGRAFT_REJECTION | 2.259591 | 0 | 0 |
|  | KEGG_TYPE_I_DIABETES_MELLITUS | 2.206412 | 0 | 0 |
|  | KEGG_SYSTEMIC_LUPUS_ERYTHEMATOSUS | 2.193989 | 0 | 0 |
|  | KEGG_CYTOKINE_CYTOKINE_RECEPTOR_INTERACTION | 2.120824 | 0 | 0 |
|  | KEGG_HEMATOPOIETIC_CELL_LINEAGE | 2.108925 | 0 | 0 |
|  | KEGG_LEISHMANIA_INFECTION | 2.107775 | 0 | 0 |
|  | KEGG_ANTIGEN_PROCESSING_AND_PRESENTATION | 2.036762 | 0 | 0 |
|  | KEGG_PRIMARY_IMMUNODEFICIENCY | 2.026308 | 0 | 0 |
|  | KEGG_AUTOIMMUNE_THYROID_DISEASE | 1.950269 | 0 | 2.70E-04 |
|  | KEGG_T_CELL_RECEPTOR_SIGNALING_PATHWAY | 1.939207 | 0 | 3.08E-04 |
|  | KEGG_ASTHMA | 1.921506 | 0 | 4.01E-04 |
|  | KEGG_NATURAL_KILLER_CELL_MEDIATED_CYTOTOXICITY | 1.921331 | 0 | 3.72E-04 |
|  | KEGG_RIBOSOME | 1.895386 | 0 | 5.00E-04 |
|  | KEGG_CHEMOKINE_SIGNALING_PATHWAY | 1.885876 | 0 | 5.63E-04 |
|  | KEGG_NOD_LIKE_RECEPTOR_SIGNALING_PATHWAY | 1.837844 | 0 | 0.001237 |
|  | KEGG_HOMOLOGOUS_RECOMBINATION | 1.831002 | 0.001319 | 0.00125 |
|  | KEGG_TOLL_LIKE_RECEPTOR_SIGNALING_PATHWAY | 1.822616 | 0 | 0.001257 |
|  | KEGG_VIRAL_MYOCARDITIS | 1.814064 | 0 | 0.001453 |
|  | KEGG_OLFACTORY_TRANSDUCTION | 1.757639 | 0 | 0.004362 |
|  | KEGG_CYTOSOLIC_DNA_SENSING_PATHWAY | 1.652213 | 0 | 0.017167 |
|  | KEGG_CELL_ADHESION_MOLECULES_CAMS | 1.583904 | 0 | 0.035867 |
|  | KEGG_PROTEASOME | 1.558324 | 0.021223 | 0.04585 |
|  | KEGG_B_CELL_RECEPTOR_SIGNALING_PATHWAY | 1.541796 | 0.003452 | 0.051865 |
|  | KEGG_JAK_STAT_SIGNALING_PATHWAY | 1.532209 | 0 | 0.054928 |
|  | KEGG_FC_GAMMA_R_MEDIATED_PHAGOCYTOSIS | 1.524488 | 0.006803 | 0.057236 |
|  | KEGG_P53_SIGNALING_PATHWAY | 1.501012 | 0.010778 | 0.069984 |
|  | KEGG_COMPLEMENT_AND_COAGULATION_CASCADES | 1.496772 | 0.017442 | 0.070192 |
|  | KEGG_CELL_CYCLE | 1.478397 | 0.0076 | 0.080965 |
|  | KEGG_GLYCOSAMINOGLYCAN_BIOSYNTHESIS_CHONDROITIN_SULFATE | 1.463306 | 0.035183 | 0.090015 |
|  | KEGG_TASTE_TRANSDUCTION | 1.462423 | 0.02994 | 0.087806 |
|  | KEGG_PRION_DISEASES | 1.439383 | 0.047194 | 0.105546 |
|  | KEGG_FC_EPSILON_RI_SIGNALING_PATHWAY | 1.431863 | 0.029748 | 0.109755 |
| ZDHHC20 | KEGG_OXIDATIVE_PHOSPHORYLATION | -2.59395 | 0 | 0 |
|  | KEGG_PARKINSONS_DISEASE | -2.28259 | 0 | 0 |
|  | KEGG_RIBOSOME | -2.2415 | 0 | 0 |
|  | KEGG_HUNTINGTONS_DISEASE | -1.87496 | 0 | 0.006 |
|  | KEGG_ALZHEIMERS_DISEASE | -1.75182 | 0 | 0.022185 |
|  | KEGG_LINOLEIC_ACID_METABOLISM | -1.73548 | 0.001808 | 0.022408 |
|  | KEGG_CARDIAC_MUSCLE_CONTRACTION | -1.72367 | 0.003617 | 0.021457 |
|  | KEGG_METABOLISM_OF_XENOBIOTICS_BY_CYTOCHROME_P450 | -1.72084 | 0.001828 | 0.019414 |
|  | KEGG_GLYCINE_SERINE_AND_THREONINE_METABOLISM | -1.63277 | 0.003868 | 0.045995 |
|  | KEGG_PROTEASOME | -1.62392 | 0.007366 | 0.04533 |
|  | KEGG_ARACHIDONIC_ACID_METABOLISM | -1.54776 | 0.007692 | 0.080684 |
|  | KEGG_STEROID_BIOSYNTHESIS | -1.52627 | 0.045627 | 0.09081 |
|  | KEGG_OLFACTORY_TRANSDUCTION | -1.50902 | 0.001701 | 0.097723 |
|  | KEGG_SYSTEMIC_LUPUS_ERYTHEMATOSUS | -1.44769 | 0.009009 | 0.149805 |
|  | KEGG_DRUG_METABOLISM_CYTOCHROME_P450 | -1.42525 | 0.034991 | 0.155572 |
|  | KEGG_RETINOL_METABOLISM | -1.40504 | 0.023857 | 0.171113 |
| ZDHHC21 | KEGG_RIBOSOME | -2.22434 | 0 | 0 |
|  | KEGG_SYSTEMIC_LUPUS_ERYTHEMATOSUS | -2.11917 | 0 | 8.61E-04 |
|  | KEGG_PROTEASOME | -1.84597 | 0 | 0.015497 |
|  | KEGG_OXIDATIVE_PHOSPHORYLATION | -1.75467 | 0 | 0.038392 |
|  | KEGG_COMPLEMENT_AND_COAGULATION_CASCADES | -1.73786 | 0 | 0.037405 |
|  | KEGG_CELL_CYCLE | -1.72219 | 0 | 0.037818 |
|  | KEGG_PRIMARY_IMMUNODEFICIENCY | -1.71802 | 0 | 0.03402 |
|  | KEGG_PARKINSONS_DISEASE | -1.67287 | 0.002759 | 0.051199 |
|  | KEGG_BASE_EXCISION_REPAIR | -1.60247 | 0.013636 | 0.097215 |
|  | KEGG_P53_SIGNALING_PATHWAY | -1.53992 | 0.014025 | 0.154275 |
|  | KEGG_GRAFT_VERSUS_HOST_DISEASE | -1.53718 | 0.021398 | 0.143447 |
|  | KEGG_PHENYLALANINE_METABOLISM | -1.52124 | 0.042003 | 0.153352 |
|  | KEGG_GLYCINE_SERINE_AND_THREONINE_METABOLISM | -1.50795 | 0.034586 | 0.159124 |
|  | KEGG_PYRIMIDINE_METABOLISM | -1.50756 | 0.006925 | 0.148284 |
|  | KEGG_CYTOKINE_CYTOKINE_RECEPTOR_INTERACTION | -1.48699 | 0 | 0.166715 |
|  | KEGG_CARDIAC_MUSCLE_CONTRACTION | -1.45042 | 0.01669 | 0.208488 |
|  | KEGG_ARACHIDONIC_ACID_METABOLISM | -1.44745 | 0.030303 | 0.189497 |
|  | KEGG_PRION_DISEASES | -1.43955 | 0.044374 | 0.191345 |
|  | KEGG_INTESTINAL_IMMUNE_NETWORK_FOR_IGA_PRODUCTION | -1.42621 | 0.043228 | 0.202277 |
|  | KEGG_DNA_REPLICATION | -1.42272 | 0.047022 | 0.198226 |
|  | KEGG_NATURAL_KILLER_CELL_MEDIATED_CYTOTOXICITY | -1.41983 | 0.010204 | 0.193496 |
|  | KEGG_HUNTINGTONS_DISEASE | -1.39466 | 0.01875 | 0.206198 |
|  | KEGG_ALZHEIMERS_DISEASE | -1.39058 | 0.024876 | 0.20515 |
| ZDHHC23 | KEGG_RIBOSOME | -2.34363 | 0 | 0 |
|  | KEGG_CYTOKINE_CYTOKINE_RECEPTOR_INTERACTION | -2.21669 | 0 | 0 |
|  | KEGG_CHEMOKINE_SIGNALING_PATHWAY | -2.12424 | 0 | 0 |
|  | KEGG_NATURAL_KILLER_CELL_MEDIATED_CYTOTOXICITY | -2.05253 | 0 | 0 |
|  | KEGG_TGF_BETA_SIGNALING_PATHWAY | -1.94197 | 0 | 0.001647 |
|  | KEGG_VASCULAR_SMOOTH_MUSCLE_CONTRACTION | -1.91079 | 0 | 0.002598 |
|  | KEGG_FOCAL_ADHESION | -1.87474 | 0 | 0.003886 |
|  | KEGG_NEUROACTIVE_LIGAND_RECEPTOR_INTERACTION | -1.79226 | 0 | 0.012234 |
|  | KEGG_ECM_RECEPTOR_INTERACTION | -1.77469 | 0 | 0.015412 |
|  | KEGG_GRAFT_VERSUS_HOST_DISEASE | -1.76079 | 0.001821 | 0.015998 |
|  | KEGG_SYSTEMIC_LUPUS_ERYTHEMATOSUS | -1.75892 | 0 | 0.015223 |
|  | KEGG_BLADDER_CANCER | -1.74429 | 0.001898 | 0.016324 |
|  | KEGG_AXON_GUIDANCE | -1.72502 | 0 | 0.018528 |
|  | KEGG_PRIMARY_IMMUNODEFICIENCY | -1.70993 | 0.003802 | 0.021279 |
|  | KEGG_LEISHMANIA_INFECTION | -1.67074 | 0 | 0.030417 |
|  | KEGG_NOD_LIKE_RECEPTOR_SIGNALING_PATHWAY | -1.65282 | 0.005396 | 0.03302 |
|  | KEGG_HEMATOPOIETIC_CELL_LINEAGE | -1.64987 | 0 | 0.032402 |
|  | KEGG_NOTCH_SIGNALING_PATHWAY | -1.62434 | 0.0125 | 0.038107 |
|  | KEGG_ARRHYTHMOGENIC_RIGHT_VENTRICULAR_CARDIOMYOPATHY_ARVC | -1.56705 | 0.007067 | 0.06108 |
|  | KEGG_MAPK_SIGNALING_PATHWAY | -1.56342 | 0 | 0.059909 |
|  | KEGG_CELL_ADHESION_MOLECULES_CAMS | -1.56092 | 0.001715 | 0.058339 |
|  | KEGG_CALCIUM_SIGNALING_PATHWAY | -1.54728 | 0 | 0.062019 |
|  | KEGG_NEUROTROPHIN_SIGNALING_PATHWAY | -1.51314 | 0.005059 | 0.082802 |
|  | KEGG_JAK_STAT_SIGNALING_PATHWAY | -1.49678 | 0.003295 | 0.090716 |
|  | KEGG_PATHWAYS_IN_CANCER | -1.48314 | 0 | 0.097112 |
|  | KEGG_LEUKOCYTE_TRANSENDOTHELIAL_MIGRATION | -1.47398 | 0.010381 | 0.101405 |
|  | KEGG_ANTIGEN_PROCESSING_AND_PRESENTATION | -1.45066 | 0.014286 | 0.118108 |
|  | KEGG_RENAL_CELL_CARCINOMA | -1.44868 | 0.036298 | 0.115756 |
|  | KEGG_TYPE_I_DIABETES_MELLITUS | -1.42898 | 0.041958 | 0.125181 |
|  | KEGG_TOLL_LIKE_RECEPTOR_SIGNALING_PATHWAY | -1.42103 | 0.017794 | 0.125236 |
|  | KEGG_ALLOGRAFT_REJECTION | -1.41414 | 0.04562 | 0.12773 |
|  | KEGG_HYPERTROPHIC_CARDIOMYOPATHY_HCM | -1.38732 | 0.038655 | 0.14551 |
|  | KEGG_DILATED_CARDIOMYOPATHY | -1.37896 | 0.032203 | 0.150412 |
|  | KEGG_GAP_JUNCTION | -1.35865 | 0.04947 | 0.155272 |
|  | KEGG_SPLICEOSOME | -1.34647 | 0.041379 | 0.165242 |
|  | KEGG_COLORECTAL_CANCER | -1.34609 | 0.041742 | 0.161705 |
|  | KEGG_T_CELL_RECEPTOR_SIGNALING_PATHWAY | -1.32634 | 0.043554 | 0.181534 |
|  | KEGG_WNT_SIGNALING_PATHWAY | -1.27218 | 0.046512 | 0.233922 |
