# Supplementary figures and images for: Bioinformatics Analysis of the Prognostic and Biological Significance of ZDHHC-Protein Acyltransferases in Kidney Renal Clear Cell Carcinoma
Source: Front Oncol. 2020 Dec 8;10:565414. doi: 10.3389/fonc.2020.565414 (PMC7753182; doi:10.3389/fonc.2020.565414)

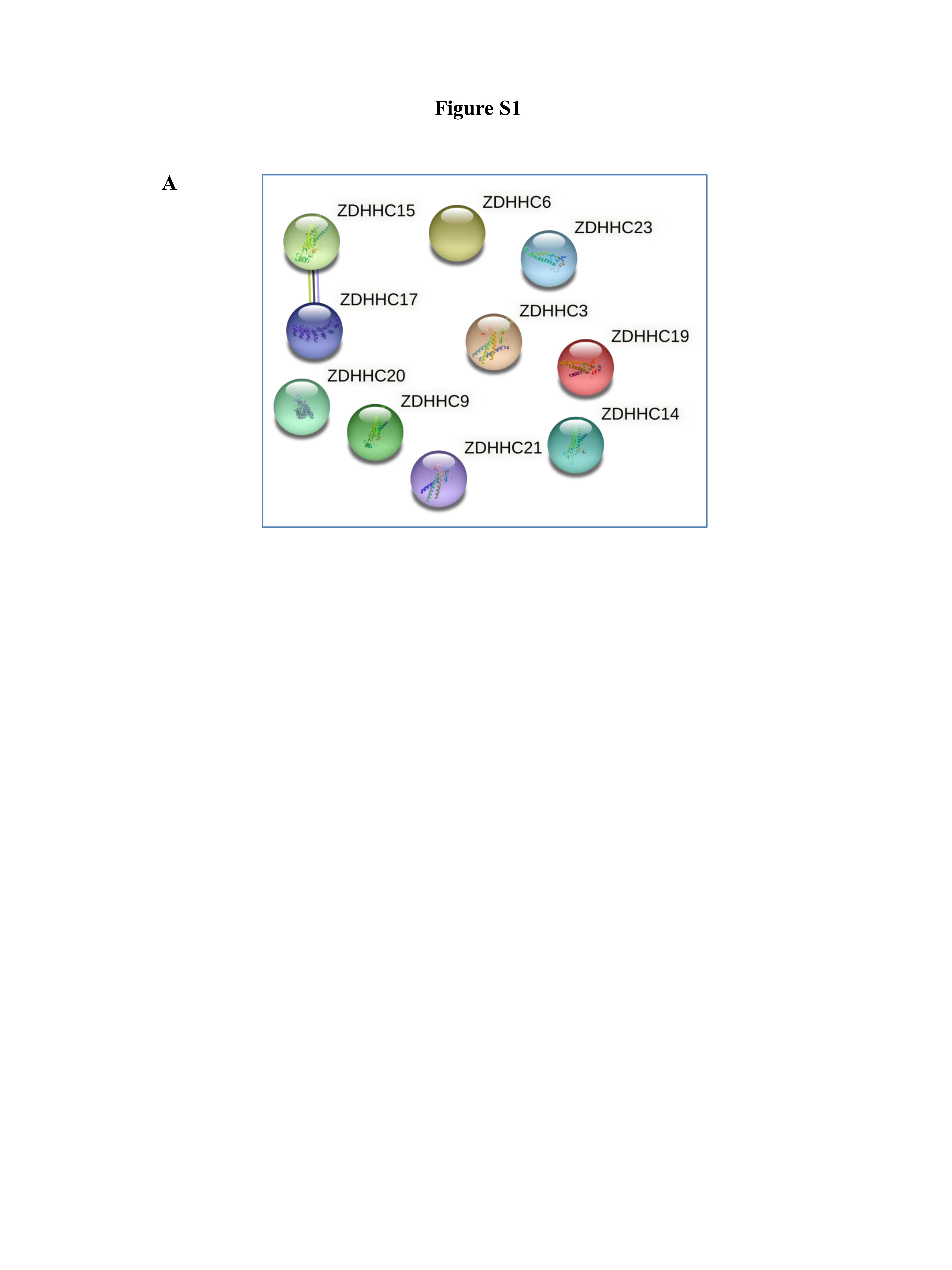

Supplement: Supplementary file 1 [file DataSheet_1.zip › Supplementary files/Figure S1.tif]
