# Supplementary material for: Bioinformatics Analysis of the Prognostic and Biological Significance of ZDHHC-Protein Acyltransferases in Kidney Renal Clear Cell Carcinoma
Source: Front Oncol. 2020 Dec 8;10:565414. doi: 10.3389/fonc.2020.565414 (PMC7753182; doi:10.3389/fonc.2020.565414)
Supplement: Supplementary file 1 [file DataSheet_1.zip › Supplementary files/Supplementary Figure titles and captions.doc]

**Figure S1. A protein-protein interaction (PPI) network constructed using the String database.**
