# Supplementary material for: Bioinformatics Analysis of the Prognostic and Biological Significance of ZDHHC-Protein Acyltransferases in Kidney Renal Clear Cell Carcinoma
Source: Front Oncol. 2020 Dec 8;10:565414. doi: 10.3389/fonc.2020.565414 (PMC7753182; doi:10.3389/fonc.2020.565414)
Supplement: Supplementary file 1 [file DataSheet_1.zip › Supplementary files/Table S1.doc]

| Table S1. The expression correlation among ten differentially expressed ZDHHCs(GEPIA). | | | | | | | | | | |
| --- | --- | --- | --- | --- | --- | --- | --- | --- | --- | --- |
|  | ZDHHC3 | ZDHHC6 | ZDHHC9 | ZDHHC14 | ZDHHC15 | ZDHHC17 | ZDHHC19 | ZDHHC20 | ZDHHC21 | ZDHHC23 |
| ZDHHC3 | 1 | 0.071 | 0.076 | 0.15 | 0.096 | 0.066 | -0.0036 | 0.084 | 0.17 | 0.48 |
| ZDHHC6 | 0.071 | 1 | 0.45 | 0.34 | 0.37 | 0.67 | -0.11 | 0.5 | 0.67 | 0.077 |
| ZDHHC9 | 0.076 | 0.45 | 1 | 0.14 | 0.3 | 0.33 | -0.12 | 0.29 | 0.42 | 0.14 |
| ZDHHC14 | 0.15 | 0.34 | 0.14 | 1 | 0.32 | 0.31 | 0.03 | 0.32 | 0.28 | -0.0077 |
| ZDHHC15 | 0.096 | 0.37 | 0.3 | 0.32 | 1 | 0.3 | -0.037 | 0.3 | 0.36 | 0.044 |
| ZDHHC17 | 0.066 | 0.67 | 0.33 | 0.31 | 0.3 | 1 | -0.056 | 0.58 | 0.74 | 0.049 |
| ZDHHC19 | -0.0036 | -0.11 | -0.12 | 0.03 | -0.037 | -0.056 | 1 | -0.075 | -0.074 | -0.033 |
| ZDHHC20 | 0.084 | 0.5 | 0.29 | 0.32 | 0.3 | 0.58 | -0.075 | 1 | 0.58 | -0.031 |
| ZDHHC21 | 0.17 | 0.67 | 0.42 | 0.28 | 0.36 | 0.74 | -0.074 | 0.58 | 1 | 0.36 |
| ZDHHC23 | 0.48 | 0.077 | 0.14 | -0.0077 | 0.044 | 0.049 | -0.033 | -0.031 | 0.36 | 1 |
